# Bump2Baby & Me+ (B2B&Me+): Protocol for a multi-country, European hybrid type 3 implementation-effectiveness study to reduce the incidence of gestational diabetes mellitus and improve maternal and child health

**DOI:** 10.1371/journal.pone.0354394

**Published:** 2026-08-31

**Authors:** Sharleen L. O’Reilly, Thérèse McDonnell, Linda Reme Sagedal, Mercedes G. Bermúdez, Florian Herrmann, Hanna Jasiak-Jóźwik, Nina Cecilie Øverby, Hilde Kristin Refvik Riise, Fionnuala McAuliffe, Helle T. Maindal, Laura Headley, Cristina Campoy, Sebastian Kwiatkowski, Ragnhild B. Strandberg, Anju Rawal, Katie Angotti, Hannah Foley, Lucy Murphy, Lin Chen, Quentin Le Cornu, Marjolein M. Iversen, Timothy Skinner

**Affiliations:** 1 School of Agriculture and Food Science, University College Dublin, Belfield, Dublin, Ireland; 2 UCD Perinatal Research Centre, University College Dublin and National Maternity Hospital, Dublin, Ireland; 3 Sørlandet Hospital Trust, Kristiansand, Norway; 4 Department of Paediatrics, School of Medicine, University of Granada, Granada, Spain; 5 Instituto de Investigación Biosanitaria de Granada, Granada, Spain; 6 Fundacion para la Investigacion Biosanitaria de Andalucia Oriental, Granada, Spain; 7 Pomeranian Medical University, Szczecin, Poland; 8 University of Agder, Kristiansand, Norway; 9 Western Norway University of Applied Sciences (Høgskulen på Vestlandet), Bergen, Norway; 10 School of Medicine, University College Dublin, Dublin, Ireland; 11 Department of Public Health, Aarhus University, Aarhus, Denmark; 12 Liva Healthcare A/S, Copenhagen, Denmark; 13 UCD School of Public Health, Physiotherapy and Sport Science, University College Dublin, Belfield, Dublin, Ireland; PLOS: Public Library of Science, UNITED KINGDOM OF GREAT BRITAIN AND NORTHERN IRELAND

## Abstract

**Background:**

Gestational diabetes mellitus (GDM) affects 1-in-7 pregnancies globally and is associated with significant short- and long-term health consequences. Although health behaviour change interventions can effectively reduce these risks, a significant implementation gap exists in translating this evidence into routine practice. Bump2Baby and Me (B2B&Me) was a mobile health (mHealth) coaching intervention provided to women at-risk of GDM from early pregnancy through to 1-year postpartum. B2B&Me Plus (B2B&Me+) aims to refine, implement and evaluate the implementation of this personalised intervention across 4 countries (Ireland, Spain, Poland and Norway) with differing health systems and population profiles.

**Methods:**

This study employs a hybrid type 3 implementation-effectiveness design using a non-randomised ABA block approach within a longitudinal cohort. Participants will be screened using the Monash machine learning GDM screening tool (MMLGDST). During the intervention (Block B), women at risk of developing GDM will be offered access to a smartphone-based coaching application featuring 1:1 synchronous sessions, asynchronous text and video messaging along with a Bluetooth-enabled weighing scale for self-monitoring. Support continues from early pregnancy through to nine months postpartum. The study’s primary objective is to evaluate reach, adoption, implementation and maintenance of the B2B&Me+ intervention programme when delivered within routine maternity care. Implementation success will be assessed using the RE-AIM framework, while secondary outcomes will assess intervention effectiveness. The study will examine population-level uptake at each site, evaluate the benefits and costs of implementation across varying contexts, and analyse how four different referral methods, randomised at the site level, affect uptake. In addition, a European implementation toolkit will be developed to provide health services with scalable strategies to bridge the evidence-to-practice gap.

**Discussion:**

This study will contribute to a growing literature on the implementation of a successfully trialled mHealth intervention in a real-world context. Understanding variation in both intervention and implementation success within a routine maternity care context across diverse settings will inform the development of an implementation toolkit to support health services in reducing the incidence of GDM and improving maternal and child health outcomes.

This study was registered with ClinicalTrials.gov (NCT07189221) on 28 August 2025 (https://clinicaltrials.gov/study/NCT07189221?cond=diabetes).

## Background

Gestational diabetes mellitus (GDM) affects approximately 1-in-7 pregnancies worldwide and is associated with significant short and long-term health consequences for both mother and baby [1,2]. Although GDM typically resolves after delivery, it remains a strong predictor of type 2 diabetes (T2D), with women who have had GDM facing a 10-fold increased risk of developing it much earlier than the average person [3]. Rates of GDM are rising, with this increase attributed to factors such as higher maternal age, excess weight and health behaviour change factors such as poor diet, low physical activity, sedentary behaviours, and stress [2,4]. Higher body mass index (BMI) is the most significant modifiable risk factor for GDM [5]. In addition, excessive gestational weight gain (GWG) is associated with a range of adverse maternal and neonatal outcomes [6–10]. Considering the consequences of GDM and excessive GWG, it is important to mitigate risk and improve maternal and neonatal outcomes.

Diet and physical activity interventions during pregnancy can effectively reduce GWG, GDM risk, and other pregnancy complications [11–13]. Systematic review and meta-analysis concluded that interventions using diet, physical exercise, metformin, and/or myoinositol compared to usual care reduced the incidence of GDM [14]. Despite greater awareness of health behaviour change’s importance, it remains challenging to maintain engagement within traditional behaviour modification programmes. Mobile health (mHealth) interventions offer a promising solution by providing scalable, cost-effective, and accessible support for health behaviour change modification. The Horizon 2020-funded Bump2Baby and Me study on 865 women at risk of developing GDM demonstrated promising results [15]. The intervention group had a lower incidence of GDM (14.0% vs. 20.0%, P = 0.027), had lower 12-month fasting glucose (5.01 vs. 5.17 mmol/L, P = 0.009), as well as lower insulin (102.09 vs. 141.17 pmol/L, P = 0.034) and triglycerides (1.69 vs. 1.81 mmol/L, P = 0.038), and higher HDL cholesterol (1.48 vs. 1.43 mmol/L, P = 0.007). Any breastfeeding at 6 weeks postpartum was higher in the intervention group (88.1% vs 81.2%, P = 0.02), as was exclusive breastfeeding at 3 months (60.6% vs 51.9%, P = 0.047). Self-weighing during either pregnancy or postpartum was associated with a reduction in BMI of 2.34 kg/m^2^ (−3.68 to −0.99) and during both periods with a reduction of 3.45 kg/m^2^ (−4.68 to −2.21) [15]. Limited evidence exists on translating this evidence into routine antenatal and postpartum care.

Guidance on delivering mHealth coaching programs or digital health interventions covering health outcomes, cost-effectiveness, and long-term sustainability are lacking [16]. Health services face a daunting task when implementing such interventions with limited resources. Staff shortages and high GDM rates compound these issues, resulting in women not receiving optimal, guideline-led care when they need it most. Therefore, greater emphasis must be placed on implementation research to ensure more coherent global evidence and the alignment of such interventions within the context of underlying system constraints [16].

A structured approach to evaluating how interventions perform when translated into routine practice rather than under controlled trial conditions is critical for research implementation. One such established framework is RE-AIM (Reach, Effectiveness, Adoption, Implementation, Maintenance) [17], which has been extensively applied across public health and health services research to evaluate intervention translation. While the areas of reach and effectiveness are commonly reported in detail, adoption, implementation and maintenance remain under-reported [18]. The B2B&Me+ project aims to address this gap by testing the real-world effectiveness of the mHealth coaching programme within regular maternity services across four European countries. Implementation in four different locations that vary greatly in terms of health systems, including national GDM screening pathways and referral processes, and demographic profile such as ethnicity and socioeconomic status, will improve the understanding and delivery of implementation. This geographical diversity will provide valuable insight on which factors are common barriers, and which are likely to be country specific. B2B&Me demonstrated good penetration and uptake within the context of a clinical trial. However, clinical trials are not designed to capture the broader service-level impact, the real-world strain on hospital resources, staff workload, and operational workflows. Therefore, to assess the challenges, advantages, and impacts associated with integrating B2B&Me+ into routine healthcare services, this study will examine population-level uptake at each location, evaluate the benefits and costs of implementation across varying contexts, and analyse how different referral methods affect uptake.

### Conceptual framework and rationale

The Exploration, Preparation, Implementation, Sustainment (EPIS) framework [19,20] is B2B&Me + ’s overarching conceptual framework, guiding the study design and structuring our understanding of the multi-level factors that influence implementation across four diverse health systems. EPIS describes four sequential phases of evidence-based practice implementation and maps these against both the outer context (national health systems, policy environments, population-level GDM burden, and digital health infrastructure across Ireland, Spain, Poland and Norway) and the inner context (each maternity site’s organisational readiness, staff capacity, existing antenatal care pathways, and attitudes towards mHealth tools). Bridging factors, particularly the consortium partnership structure and the involvement of Liva Healthcare A/S as the mHealth platform provider, connect these contexts and support consistent delivery across sites. The B2B&Me + mHealth coaching programme constitutes the innovation factor, characterised by the evidence base from the original B2B&Me RCT, the Monash gestational diabetes screening tool (MMLGDST), and the personalised health coaching platform [21].

The EPIS phases map directly onto B2B&Me+ study activities. *Exploration* encompasses the original RCT evidence, the implementation gap in translating mHealth behaviour change interventions into maternity care, and this protocol development. *Preparation* is operationalised through pre-implementation contextual mapping at each site, characterising inner and outer context barriers and facilitators prior to study commencement, and informing site-level preparation including staff training and mHealth app adaptations. *Implementation* encompasses the study itself, a non-randomised ABA block design, where women are screened using the MMLGDST and invited to participate in the B2B&Me + mHealth coaching programme using one of four randomised referral strategies within the intervention period (Block B). This block is then compared with two usual care blocks before and after. The rationale for comparing referral approaches (point-of-care referral and information leaflet, both with and without active follow-up) was informed by prior evidence that active follow-up strategies yield higher proportional enrolment while more passive strategies achieve greater absolute penetration [22]. *Sustainment* is addressed through the co-design of a European implementation toolkit, which will translate study learnings into scalable guidance for health services considering future adoption of the programme.

Normalisation Process Theory (NPT) [23] complements EPIS by providing the study’s qualitative analytical framework. Interviews and focus groups with health coaches, site coordinators, and clinical leads will be structured around NPT’s four constructs (coherence, cognitive participation, collective action, and reflexive monitoring) to ask what work is needed and by whom to make this innovation become routine, embedded and sustained within everyday maternity services. Findings from this analysis will directly inform the implementation toolkit, supporting understanding of the conditions under which B2B&Me+ might be normalised into routine maternity care in the future.

### Study aims

Aim 1 (Primary — Referral Methods): To compare participation rates achieved by point-of-care versus information leaflet referral strategies among MMLGDST-identified at-risk women, hypothesising that point-of-care approaches will yield higher proportional participation [22].

Aim 2 (Primary — Implementation): To evaluate the reach, adoption, implementation and maintenance of B2B&Me+ across four European maternity services using the RE-AIM framework, with EPIS guiding exploration of the inner and outer context factors that explain variation across sites.

Aim 3 (Secondary — Effectiveness): To assess the effectiveness of B2B&Me+ on maternal and neonatal outcomes, hypothesising lower GDM incidence, reduced gestational weight gain, and improved breastfeeding outcomes in the intervention block compared with usual care, consistent with the B2B&Me RCT [15].

Aim 4 (Exploratory — Implementation Experience): To explore the implementation experience of health coaches, site coordinators and clinical leads using NPT as an analytical lens, generating evidence to inform the co-design of a European implementation toolkit.

## Materials & methods

### Project design

**Fig 1:**
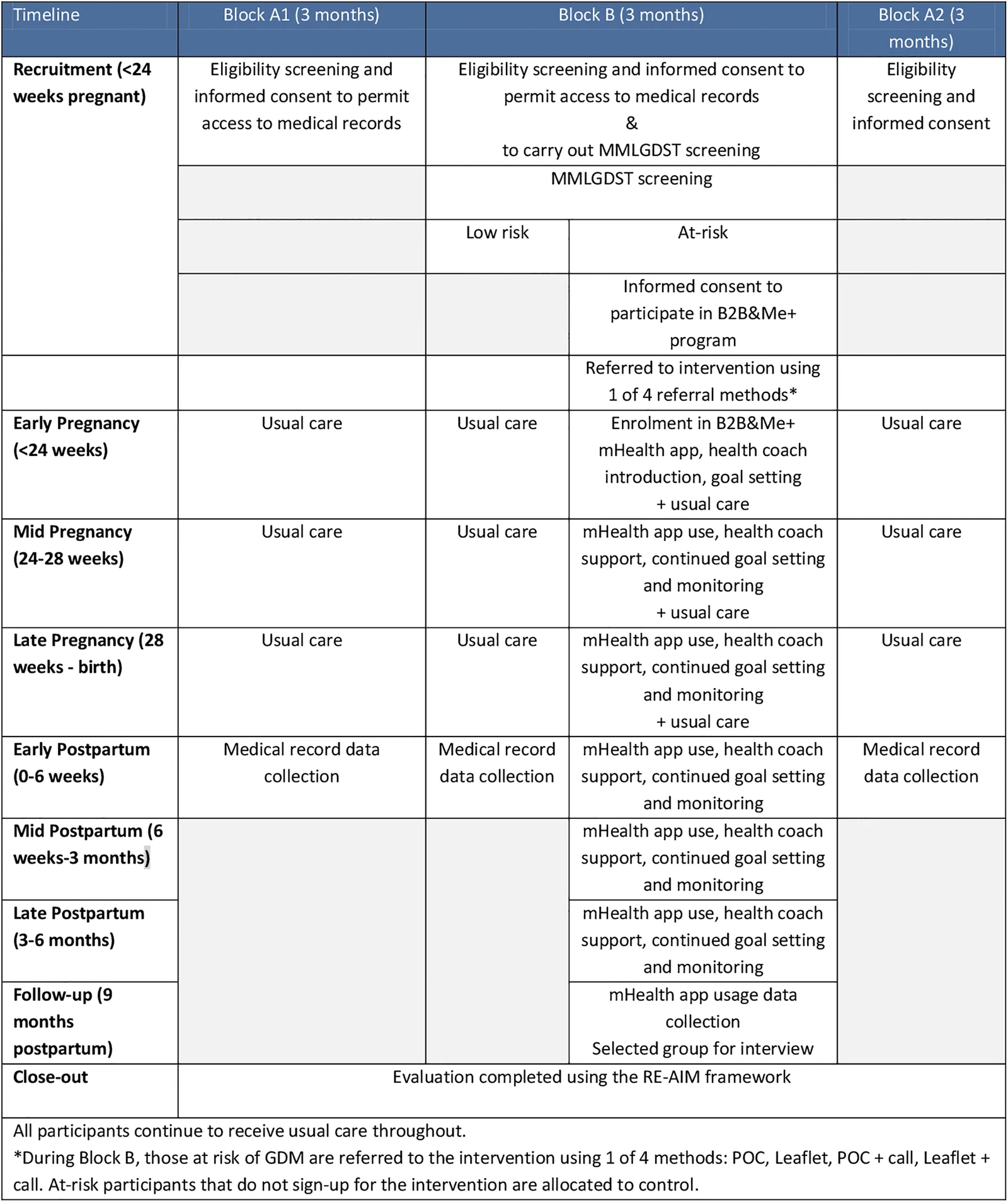
Adapted SPIRIT 2025 schedule of enrolment, intervention and evaluation.

This protocol is reported in accordance with the SPIRIT (Standard Protocol Items: Recommendations for Interventional Trials) 2025 statement (Fig 1 and Supplementary file1) [24] and the Standards for Reporting Implementation Studies (StaRI) (Supplementary file 2) [25].

This is a hybrid type 3 implementation-effectiveness study using a non-randomised, ABA intervention design nested within a longitudinal cohort. As a type 3 hybrid study, focus is primarily on implementation outcomes, with secondary outcomes assessing intervention effectiveness [26]. This study was registered with ClinicalTrials.gov (NCT07189221) on 28 August 2025 (https://clinicaltrials.gov/study/NCT07189221?cond=diabetes).

**Fig 2:**
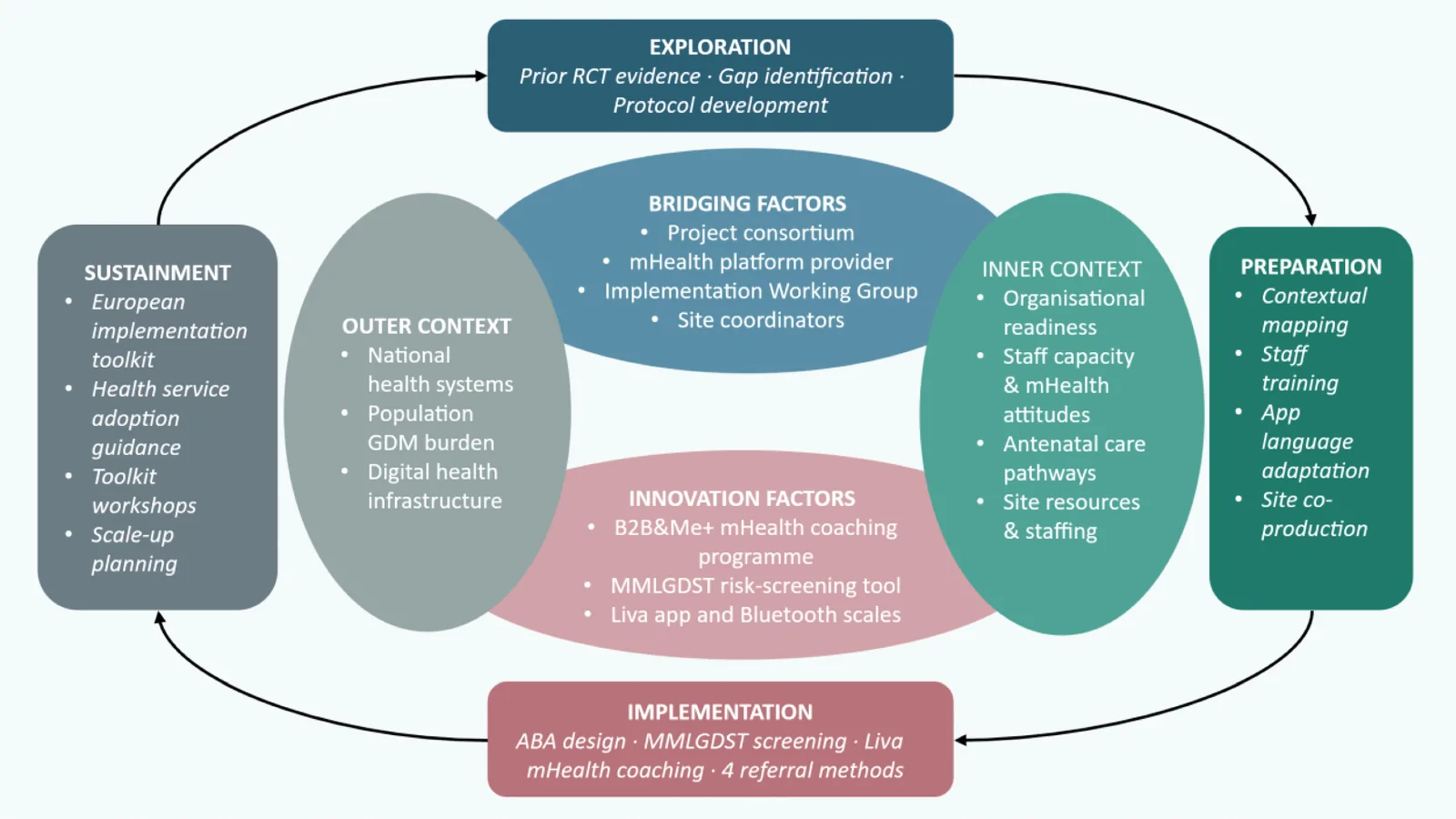
EPIS framework applied Bump2Baby and Me Plus mHealth coaching intervention (Adapted from: Aarons et al. (2011) Adm Policy Ment Health; Moullin et al. (2019) Implement Sci [19,20]).

The EPIS phases guide the study design and map directly onto B2B&Me+ study activities (Fig 2).

### Implementation strategy

The study will compare the delivery of the MMLGDST and mHealth coaching referral during a 3-month intervention block (B) with two 3-month blocks of usual care, before and after the intervention (A blocks) [21]. Recruitment to the study at all sites commenced on 1^st^ December 2025 and is scheduled to conclude on 31^st^ August 2026 (Table 1). All participants will be followed through-out pregnancy and those in the intervention will also be followed-up until 9-months postpartum. Data collection is expected to be completed by November 2027, and results are expected by May 2028.

**Table 1:** Date range for participant recruitment and follow-up.

Stage (All sites)	Block A1	Block B	Block A2
	Control	Intervention	Control
***Recruitment:***			
Commencement	1^st^ December 2025	1st March 2026	1^st^ June 2026
Completion	28^th^ February 2026	31^st^ May 2026	31^st^ August 2026
***Follow-up:***			
**Antenatal period**			
Commencement	1^st^ December 2025	1^st^ March 2026	1^st^ June 2026
Completion	31^st^ August 2026	30^th^ November 2026	28^th^ February 2027
Birth*			
Commencement	1^st^ March 2026	1^st^ June 2026	1^st^ September 2026
Completion	31^st^ August 2026	30^th^ November 2026	28^th^ February 2027
Postpartum period**			
Commencement	1^st^ March 2026	1^st^ June 2026	1^st^ September 2026
Completion	16^th^ October 2026	31^st^ August 2027	16^th^ April 2027

*Recruited at gestation <24 weeks. Based on estimated date of delivery (36 weeks+).

**Postpartum follow-up 9 months for intervention participants. Limited to date of postnatal discharge for control group (Block A and Block B (not in intervention)).

All participants will complete a baseline questionnaire and provide written informed consent to access medical records. While Block A participants will not undergo risk screening, their GDM risk status will be determined from the data provided. Within Block B, women attending the antenatal clinics across the four sites will be recruited either in-person or by phone. During the intervention block (B), women will provide written informed consent to be screened using the MMLGDST [21]. Those screening positive to being at-risk of GDM will be provided with information about the B2B&Me+ programme and invited to provide additional written informed consent for the intervention.

Pregnant women attending their first/early antenatal appointments at participating sites will be assigned to a block based on the date of their visit. Gestation must be less than 24 weeks at enrolment; participants must be aged 18 years or older and able to provide informed consent. Women with a multiple pregnancy (twins, triplets, etc.), existing diabetes (Type 1 or Type 2) or early GDM are excluded. To be considered eligible for the intervention (Block B only), women must be identified as at-risk for GDM using the MMLGDST and must also own a smartphone. Additional exclusion criteria apply for the intervention group:

- Participation in another health behaviour change intervention study during pregnancy
- Inability to understand the language of the intervention (English in Ireland, Spanish in Spain, Norwegian in Norway, Polish in Poland)
- Severe mental illness, drug or alcohol abuse that would impair ability to participate
- Cancer (not in remission)
- Myocardial infarction in the last three months
- Not owning a smartphone capable of hosting the intervention app.

### Context

The assessment of context is important for implementation evaluation. The study will be conducted in four maternity hospitals across Europe:

1. National Maternity Hospital, Dublin, Ireland
2. University Hospital San Cecilio, Granada, Spain
3. Sørlandet Hospital Trust, Kristiansand, Norway
4. Pomeranian Medical University Hospital, Szczecin, Poland

These sites were selected to represent diverse healthcare systems, populations, and models of maternity care across Europe. Contextual barriers and facilitators that are linked to different roles, tasks, education, and skills of health and care professionals will be considered within the project and have their own dedicated work package. At the outset of the project, researchers will assess knowledge, attitudes and barriers that exist within the four study hospital sites to adopting the intervention and develop a tool to speed up this implementation process into the future. Staff within each hospital will be surveyed and interviewed to see what their awareness level is of related services that are available and the work associated with supporting women at risk of developing gestational diabetes. On completion of the intervention, a subset of intervention participants from each site will be invited to participate in an interview to explore their intervention experience as well as implementation barriers and enablers. Researchers involved in the recruitment and management of the study and mHealth coaches will also be invited to take part in an interview/focus group for the implementation analysis of the project.

### Intervention referral

Within Block B, different referral methods will be tested every 2 weeks for women assessed as at-risk of GDM and meeting the eligibility criteria:

i Point-of-care (POC) active referral: programme offered either in-person or by phone if the woman agrees to a phone consultation after the initial antenatal appointment. The participant is supported to start the sign-up process for the B2B&Me + mHealth coaching app and the VitaDock+ Bluetooth scales app during this contact time.
ii POC with follow-up phone call: As above, plus follow-up call within 48 hours to support sign-up.
iii Leaflet provided: programme offered either in-person or by phone if the woman agrees to a phone consultation after the initial antenatal appointment. On enrolment, the participant is provided with an information leaflet about the sign-up process for both apps.
iv Leaflet with follow-up call: As above, plus follow-up call within 48 hours to support sign-up.

The referral allocation will be site-stratified and randomised. The randomisation sequence will be generated by the project manager who is not involved in participant recruitment using computer-generated random numbers. Site coordinators will enrol participants to the appropriate intervention pathway based on the randomisation schedule. The sequence will be stored securely by the project manager and revealed to each site no more than two working days before the commencement of each 2-week randomisation block. The active follow-up versus no follow-up comparison will be evaluated as a 2 × 2 factorial main effect and treated as exploratory if participation rates fall below base case estimates.

### The intervention

Women consenting to participate in the intervention will be enrolled in the B2B&Me + mHealth coaching app and asked to schedule an appointment with a health coach through the B2B&Me + app. Participants will be provided with Bluetooth-enabled weighing scales, Medisana BS 444 connect [27], for regular weight self-monitoring. Weight data will be transferred to the VitaDock+ weighing scale app, which in turn is shared with the Liva Healthcare app for coaching purposes (weight data is shared only if the participant provided the necessary permissions between the apps).

### mHealth coaching

**Fig 3:**
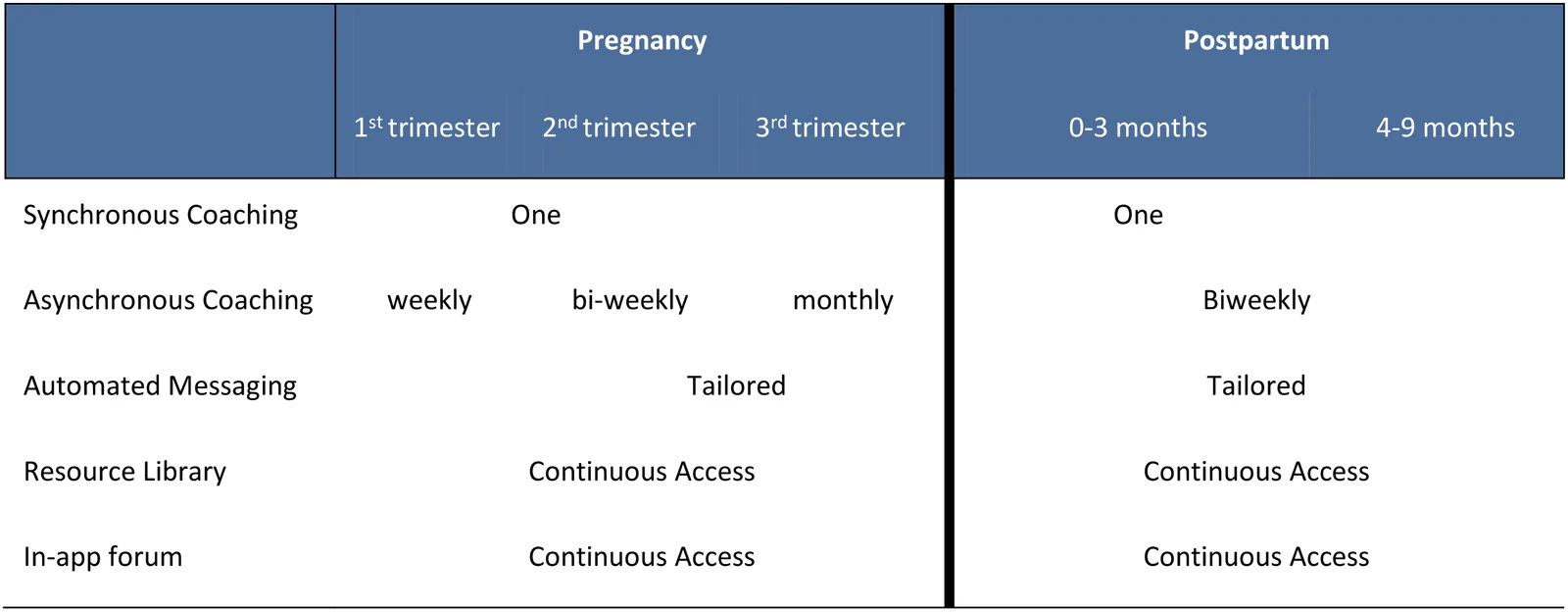
Intervention Delivery.

Both synchronous and asynchronous coaching sessions will be delivered through the B2B&Me + mHealth coaching app during the intervention (Fig 3). Two synchronous sessions will occur, the first at enrolment and second around 8 weeks postpartum. If diagnosed with GDM during pregnancy, a third session to review any health behaviour change goals to align with their diabetes management plan will be offered. Asynchronous coaching messages will be delivered according to a pre-defined schedule using a combination of both text and video. The asynchronous sessions will be weekly for the first four intervention weeks, fortnightly for two months and then monthly until birth. The postpartum sessions will be bi-weekly to support the participant to ease back into the mHealth coaching contact after birth and continue until 9 months postnatal. Session delivery will be adapted according to participant preferences.

### mHealth resources

As part of the asynchronous messaging, participants will also receive educational resources from their health coach. This content will cover topics within healthy eating, physical activity, emotional wellbeing, breastfeeding, and best practice formula feeding. Each mHealth coach will continuously assess what content is relevant before sending it to the participants, and this content will also be freely available in the static library within the B2B&Me + app. Follow-up messages prompting the participant to engage with the content and follow through with goals they have set for themselves will be sent through the app. The mHealth coaching platform records which of the coaching messages, educational content and goals are accessed, and this data will be used to determine the total number of messages received by message content (information, motivational, reminder). This will be used to generate an exposure index for each message type, for which the denominator will be the highest number of messages received by any one individual participant. Reminders to set goals and motivational messages once completed will be used to encourage engagement. Push notifications will be sent through the app signposting participants to additional content available to them during the weeks where no asynchronous coaching occurs. Participants will also have access to a monitored chat forum through the app to engage with other participants.

The lead development and training personnel at Liva Healthcare, alongside key study staff, will conduct the mHealth coach training. The coaching model is underpinned by a theoretical framework that draws from Social Cognitive Theory [28], Self-Determination Theory [29], and the Transtheoretical Model of Behaviour Change [30]. In addition, coaches utilise Behaviour Change Technique (BCT) taxonomy, specific Motivational Interviewing Technique (MIT) taxonomy and Motivational Behaviour Change Technique (MBCT) taxonomy in order to support the participant in identifying and working towards their health and lifestyle goals. The standard Liva mHealth coach training takes a four-pronged approach, inclusive of:

1. Patient communication: including full training on the Liva coach dashboard and patient app interface.
2. Behaviour change skills: coaches are recruited with health professional backgrounds because they already have skills in supporting people to engage in behaviour change. The refresher course, and coaching handbook provided aims to build on this in line with the specific behaviour change skills used throughout Liva coaching structures.
3. Practicalities: including time management topics, remote team support and communications.
4. Patient safety and clinical pathways: medical and mental health governance structures and escalation pathways, any intervention-specific requirements.

In addition to this coaching training, all mHealth coaches will receive specific training related to the prevention and management of GDM, identification of and support for participants with postnatal depression, breastfeeding and infant feeding, infant development, and managing health for diabetes prevention.

All participants will continue to receive routine antenatal and postnatal care according to local guidelines. There are no restrictions on concomitant care or interventions, except participation in other research studies involving health behaviour change interventions during pregnancy.

Participants may withdraw from the intervention at any time without giving a reason and without any impact on their usual care. The intervention may be discontinued or modified for a participant if the participant experiences significant distress related to the intervention, develops a condition that would make continued participation inappropriate (e.g., severe pregnancy complications requiring bed rest), loses access to a compatible smartphone, or the health coach identifies concerns requiring referral to specialist mental health services.

### Adverse events

This is a low-risk intervention (physical activity and healthy eating mHealth coaching). However, adverse events (AEs) may include technology-related issues (e.g., data privacy concerns), health coaching interactions (e.g., inappropriate advice), increased anxiety or stress associated with the intervention, or physical health events (e.g., injuries during increased physical activity). Serious adverse events (SAEs) are defined as:

i serious (resulting in: death, life-threatening episode, inpatient hospitalisation or prolongation of hospitalisation, persistent disability or incapacity, congenital abnormality or any other medical event deemed significant by the investigator);
ii can be attributed, i.e., *related* to the intervention; and
iii unexpected

If AEs occur, where required, care will be provided to the participant and local reporting and management procedures will be adhered to. Serious adverse events (SAEs) must be reported to the Research Ethics Committee (REC) within a week of the investigative team being informed.

At the conclusion of the study, intervention participants will receive information about continuing healthy habits and when to seek medical advice. They will also be provided with resources on local support services for maternal and child health.

### Primary outcomes: Implementation

The implementation process will be evaluated using the RE-AIM framework (Table 2) [18]. This framework ensures this study will collect the data necessary and adopt measures to identify the translatability and health impact of the intervention, balancing the emphasis between internal and external validity, and between individual and organisational level impact. Reach and effectiveness are individual-levels of impact, whereas adoption and implementation are organizational-levels of impact. Maintenance/sustainment can be both an individual- and an organizational-level of impact. It is pertinent to evaluate both levels because each provides valuable independent information of intervention impact [31].

**Table 2:** RE-AIM dimensions and indicators.

RE-AIM dimension and definition	Indicator
**Reach** Penetration and participation in B2B&Me+	% women (Block B) risk assessed out of the eligible hospital population (penetration) % women eligible agreeing to participate in the trial (Block B) (trial participation) % women completing health coaching sessions (health coaching participation) App and platform analytics for intervention women (mHealth engagement) Characteristics of participants by each site compared with the hospital population (representativeness) Delivery of each referral method
**Effectiveness** B2B&Me+ impact on outcomes	*Block A At-Risk v Block B Intervention (At-Risk)* % women diagnosed with GDM % women with any complications recorded Change in BMI at delivery % women delivered via each mode of birth Placenta weight recorded (g) Infant birth weight (g), length (cm), and head circumference (cm) *Block B – intervention* Change in BMI at 9 months postpartum Duration of breastfeeding Change in physical activity/diet/sleep/ wellbeing at 9 months postpartum
**Adoption:** Site and individual level willingness to initiate B2B&Me+	*Site adoption:* Number of site training sessions delivered Attitudes and engagement assessment of key informants and general staff *Health coach adoption:* Number of health coach training sessions delivered % retention of health coaches
**Implementation:** Fidelity of B2B&Me+ to protocol and uptake of the intervention Cost-effectiveness analysis	% participants who receive a minimum number of health coaching sessions % participants who record goals as part of health coaching Maintenance of health behaviour change goals achieved *Program fidelity:* % scheduled contacts completed Protocol deviations: missed/delayed contacts, technical issues, deviations logged *Implementation challenges:* Initial exploratory findings developed through interviews and focus groups with site leads & staff, and review of implementation working group minutes Interviews with staff, site leads and health coaches on the implementation process. *Cost-effectiveness analysis (site-level)* Total intervention cost; Total implementation cost; incremental cost to achieve penetration and participation in early pregnancy GDM screening; incremental cost to achieve recruitment to the intervention, distinguishing between the 4 referral methods; incremental cost to achieve engagement with the intervention, including extent of engagement (dosage); incremental healthcare cost per case of gestational diabetes avoided, gestation weight gain and BMI, and other maternal and infant outcomes
**Maintenance:** Extent to which B2B&Me + has the potential for long-term effects	*Potential for future implementation:* Budget Impact Analysis: Base case Ireland, adapted for Poland, Norway and Spain.

Note: Not all these data may not be available at all sites due to local data capture practices.

Qualitative data from all interviews and focus groups will be analysed using NPT as the analytical framework to explore the mechanisms underlying RE-AIM implementation outcomes across sites.

### Secondary outcomes: Intervention effectiveness

Secondary outcomes will assess the effectiveness of the intervention relative to usual care only for the following outcomes:

- Change in maternal weight and BMI (gestational weight gain)
- GDM incidence
- Breastfeeding initiation and on discharge, & breastfeeding duration
- Diet quality
- Physical activity
- Rates of adverse birth outcomes (pre-eclampsia, preterm birth, birth weight ≥ 4000g, birth trauma, neonatal respiratory distress, phototherapy, stillbirth/neonatal death, NICU admission or shoulder dystocia, caesarean section/interventions)
- Healthcare utilisation

### Data on participants

Data will be collected from all participants using a questionnaire administered at baseline recruitment. Medical records will be used to validate this information and to track the participants throughout the pregnancy, birth, and up to postnatal discharge. Data will also be sourced from the mHealth app for those within the intervention, which will include health and behaviour outcomes, and statistics on their engagement with the app and the health coach (Table 3). Should a participant discontinue with the intervention, all outcome data will continue to be collected in accordance with the consent provided.

**Table 3:** Data collected from participants.

Data Collected from Participants		
**ALL PARTICIPANTS**		
**Data collected at Baseline** **(Questionnaire & cross-check against medical records)**		
**Demographic characteristics**	**Clinical information**	
• Age • Ethnicity • Educational attainment • Relationship status	• Gestational age at inclusion • Relevant medical history including PCOS diagnosis • Obstetric history (gravidity, parity, previous complications) • Prior GDM screening and results • Anthropometric measurements (weight and height) • Blood pressure	
**Data extracted from Medical Records**		
**Antenatal Assessments**	**Birth and postnatal**	
• 20-week scan details • Glucose tolerance testing (<24 weeks & 24–28 weeks) • Gestational diabetes diagnosis • Antenatal visit record • Admissions (unplanned/planned) • GDM treatment type	• Gestational age at delivery (weeks) • Weight of woman (collected closest to delivery) • Delivery method, including any medical interventions • Birth complications, including admission to the NICU, special care nursery and/or neonatal hypoglycaemia • Placental weight • Apgar score • Newborn weight • Newborn length • Newborn head circumference • Breastfeeding initiation • Breastfeeding status on discharge • Lactation support services accessed • Blood results for anaemia investigations and glycaemia status • Healthcare utilisation (appointments, admissions – as captured on hospital records)	
**PARTICIPANTS IN INTERVENTION ONLY**		
**Assessments recorded in the B2B&Me + app**		
**At recruitment**	**Periodically throughout the intervention** **(as uploaded by participant)**	**9 months postpartum**
• FIGO nutrition checklist scores • WHO-5 • MMLGDST classification	• Weight (recorded using the Bluetooth scales provided) • Steps/Activity tracking	• FIGO nutrition checklist scores • WHO-5
Adherence to the intervention will be monitored through app usage metrics, completion of self-monitoring activities, and engagement with the health coach.		

Note: Not all these data may not be available at all sites due to local data capture practices.

Data collected will be entered on standardised Case Report Forms (CRFs) within an online, secure platform, with data stored on a secure server located at the lead institution. The data entry platform will be accessible to research staff through the use of individual log-ins. Local researchers will have access restricted to their own site. Data captured in the database will be pseudonymised, with all patients assigned a Study ID. Each CRF has an attaching data dictionary and data entry with follow Standard Operating Procedures (SOPs) for each form.

### Economic evaluation

As a hybrid type 3 study, the cost effectiveness of both running and implementing the intervention will be assessed for sites individually and collectively as follows:

i The incremental cost of the intervention compared to usual care per health outcome achieved (see above)
ii The incremental total cost (intervention + implementation) per health outcome achieved.

#### Intervention cost-effectiveness analysis.

The initial cost-effectiveness analysis (CEA) will calculate gains in treatment effect and costs of the intervention and compares the treatment effects and costs to the comparator, usual care. The product of the cost-effectiveness analysis is an incremental cost-effectiveness ratio (ICER) which expresses the additional costs per additional outcome gained for the intervention compared to usual care for pregnant women at risk of gestational diabetes.

#### Intervention implementation CEA.

The capture of costs must be sufficiently comprehensive to ensure the cost of fully replicating the intervention is measured. As the treatment and reporting of costs and outcomes in this project must facilitate implementation to other settings – scaling up (to similar organizations or systems) and scaling out (to different types of settings and locations), reporting will focus on replicability through full transparency in results [32]. Implementation costs include intervention replication costs that would be required by those adopting the program [33], costs related to intervention tailoring or adaptation, costs to engage practitioners and participants, and any other costs related to implementation strategies such as training, supplies and labour costs for activities such as provider coaching or consultation. Costs and outcomes will be reported at site-level, with the local contextual factors likely to influence results identified. Intervention costs (e.g., coaching support, app costs, etc.) will be captured, as will implementation costs, many of which are activity-driven based on time taken to complete implementation tasks [34]. Research-specific activities that are essential to designing and developing the original implementation will be excluded [32,35]. Participant and staff perspectives on the acceptability, feasibility and usefulness of the intervention and its fidelity will be gathered using focus groups and/or interviews in order to better understand the full story of the costs and outcomes of implementation. Results from the economic evaluation will inform more widespread scale-up of this intervention.

#### Referral CEA.

Comparative economic evaluation of implementation strategies provides critical information for payers, policymakers, and providers to make informed decisions if specific strategies are an efficient use of scarce organizational resources [36]. A systematic evaluation of each implementation strategy and the costs of achieving them will be undertaken [34]. Cost-effectiveness analysis will assess variation in recruitment between each of the four referral methods. These referral methods vary in their resource requirements: leaflet versus point of care (POC) referral, and both options accompanied by a follow-up phone call. Capturing implementation costs will involve the completion of a tracking form (Supplementary File 3: Table A4) by each site that will record the time spent and costs incurred recruiting women to the intervention, distinguishing between research costs (e.g., obtaining consent), intervention and implementation costs common to all four recruitment methods (e.g., completing the risk assessment), and time and costs specific to each of the four recruitment strategies. To minimise the burden of data collection, the template will be completed for a sample of five consecutive participants recruited to the intervention per 2-week block. Site-specific pay scales for staff involved in the delivery and/or implementation of the intervention in a real-world context will be used to estimate activity-based estimates of the cost of conducting each specified step of the four recruitment strategies. The incremental cost of implementing each of the four recruitment strategies, with *Leaflet Only* as the base case, will then be assessed against the relative recruitment success.

#### Budget impact analysis.

A budget impact analysis (BIA) will estimate changes in the expenditure incurred by the healthcare system after adoption of B2B&Me + . This approach suits the implementation science context where financial factors are central to whether programs and services are adopted and sustained [36]. Key questions include how effective is the implementation of the intervention in reaching the target population, the staffing and resources required to meet the new demand for services, and the budget required going forward [32]. The BIA takes a broad perspective to include the entire healthcare organisation as beneficiaries and assess the downstream budget impacts: costs saved via preventive care [37]. The Irish Healthcare system will be the initial base case for the BIA.

### Sample size

Observed data from Block A1 across all four sites confirm that 65% of women screened using the MMLGDST are classified as at-risk for GDM, yielding approximately 195 eligible women per block per site. Of these 20% are anticipated to decline intervention participation, which is roughly half the number of women declining participation for the B2B&Me RCT where randomisation to control arm existed. On this basis, we expect to recruit approximately 150 intervention participants per site and 600 across all sites (Table 4). The at-risk women identified during the two usual care blocks (Blocks A1 and A2) constitute the comparison group, yielding n = 1,560 comparators (195 at-risk × 2 blocks × 4 sites). For the primary clinical effectiveness outcome (GDM incidence), we powered based on observed rates from the B2B&Me RCT, where GDM was diagnosed in 14.0% of intervention participants (53/379) compared with 19.7% of control (75/380; p = 0.034) [15]. With n = 600 intervention and n = 1,560 comparison group, the study will have 88.4% power to detect a difference of this magnitude at a two-sided significance level of 0.05, with an acceptable false negative rate of 0.116 [38].

**Table 4:** Expected participant recruitment.

	Block A1	Block B				Block A2
Weeks	1–13	14–25				26- 38
Attending/approached (A) (first visit)	1,920	1,920				1,920
Recruited (B): Risk Calculated/ Assessed*	1,200	1,200				1,200
At-Risk (C) **	780	780				780
		**At each site** **(At-Risk n = 195,** **recruited to intervention n = 150)**				
**Referral Method¥**		**1**	**2**	**3**	**4**	
Invited to participate (C)**		65	65	32	33	
Agreed to participate in intervention (D)***		50	50	25	25	

Notes:

No data on women attending and not risk assessed.

¥ Referral Methods 1 & 2 run for 4 weeks each, while 3 & 4 run for 2 weeks each.

*/**/*** Patient level data.

* Block B women are risk assessed. Risk status of Block A women is calculated based on data collected but women do not undergo a risk assessment.

** Number of women deemed at-risk per MMLGDST. For Block B, all are invited to participate in the intervention.

*** Consenting to participate in the intervention.

The referral method comparison is structured as a 2 × 2 factorial design, with referral mode (point-of-care versus information leaflet) and active follow-up (follow-up phone call versus no follow-up call) as the two independent factors. Anticipated participation rates are derived from Porter et al. (2021), who reported pooled participation rates of 46.6% for point-of-care approaches and 11.5% for leaflet approaches across a comparable hybrid type 3 referral methods study in primary care [22]. For the primary referral method comparison (point-of-care versus leaflet), applying a conservative 50% attenuation of these rates to account for population and setting differences, and a design effect of 1.5 to account for clustering by site, a minimum of 95 women per group is required to detect this difference with 80% power at α = 0.05. With approximately 520 women anticipated in the point-of-care group and 256 in the leaflet group across four sites, this comparison is robustly powered under all scenarios tested. For the secondary referral method comparison (active follow-up versus no follow-up), Porter et al. observed pooled participation rates of 22.5% and 12.5% respectively. Applying a design effect of 1.5, a minimum of 338 women per group is required at base case rates, with approximately 388 available in each group. This comparison is adequately powered under base case assumptions; however, if participation rates fall below those observed by Porter et al., findings from this comparison will be treated as exploratory. Results will be reported with 95% confidence intervals throughout to support interpretation irrespective of formal significance.

### Statistical analysis

#### Primary analysis.

Penetration and participation are important measures for the assessment of implementation. Participation is calculated as the number of participants recruited to the intervention (D) expressed as a proportion of those eligible and invited (C), while penetration is calculated based on the number of participants screened (B) expressed as a percentage of those attending the clinic and eligible for screening (A). The overall difference in penetration between the intervention and control groups and difference in participation rates across the four referral methods will be assessed. While penetration is not directly related to referral method, there may be a spillover staffing effect due to the level of resource taken to recruit to the intervention being higher for POC than leaflet recruitment, resulting in less time available for staff to conduct assessments. Therefore, penetration will also be assessed by referral method. The level of engagement by intervention participants will also be assessed, as defined by the extent of engagement with the health coach and the app. Chi-square tests will be used for categorical data and t-tests or ANOVA for continuous data.

#### Secondary analysis.

Secondary analysis will compare the effectiveness of the intervention across different blocks (A1 & A2 (at-risk) vs B (intervention)) and by site. This will be analysed using chi-square tests for categorical data and t-tests or ANOVA for continuous data. Non-normally distributed data will be analysed either after log transformation or with non-parametric methods. Linear regression analysis will account for potential confounding variables. Dichotomous outcomes will be analysed using logistic regression. Outcomes with multiple measurements at pre-defined time points will be analysed using mixed models with generalised estimating equations (GEE), with either a linear or a logistic link function, depending on the nature of the outcome.

Missing data will be handled using multiple imputation techniques where appropriate, with sensitivity analyses used to assess the impact of missing data assumptions. Pattern-mixture models will be used to explore potential non-random missing data mechanisms [39]. Sensitivity analyses will also be conducted to assess the robustness of findings to different analytical approaches and assumptions.

#### Subgroup analyses.

Subgroup analyses will be conducted to examine potential effect modifiers, including maternal age, pre-pregnancy BMI categories, parity (nulliparous vs multiparous), socioeconomic status, site/country differences, referral method (within Block B).

### Sustainment

The key beneficiaries of the project outputs are women and their infants, healthcare professionals and policy and healthcare decision makers. Exploitation of B2B&Me+ results by key stakeholders will be ensured through a range of strategic engagement and exchange activities to translate research results and approaches. The ambition of B2B&Me + is to move knowledge outputs into implementable solutions within the project duration. The project website, social and traditional media campaigns will target a broad audience. Key results of relevance to policy and decision maker stakeholders will be promoted through two-way dialogue engagement in implementation workshops where project results, including the economic analysis, and implications for policy will be discussed and shared.

On conclusion of the analysis, a European implementation toolkit will be developed to provide health services with scalable strategies to bridge the evidence-to-practice gap. The toolkit will be co-designed with all key stakeholders and aims to support healthcare services and systems looking to implement and scale up the B2B&Me+ intervention. It is planned to contain a road map on stakeholder engagement across management and frontline staff, project governance recommendations, training resources and implementation and delivery framework, recommendations and resources targeting staff, women and managers (including resources required and anticipated savings). Measurement of fidelity, adaptability and evaluation will also be covered, as will strategies to link to audit and feedback with routine clinical data sets for ongoing evaluation and improvement.

An important component of this study is the sharing of data among the collaborating sites in B2B&Me+ for the benefit of others. To facilitate this, ethical agreements include permissions to share anonymised data collected as part of this project. At the project end once analysis is complete, anonymised data with supporting data dictionary will be placed in a local/national repository for use by others according to permissions granted and procedures of individual partners.

### Project management

The Project Coordinator (PC) from the lead institution (NUID UCD) is responsible for the strategic leadership of the consortium, providing guidance, direction and steering the project. The PC leads the Project Management Team (PMT) in Dublin, which includes a dedicated Project Manager. The key tasks of the PMT include day-to-day co-ordination and operational management, ensuring the quality and consistency of technical and contractual aspects of the study, financial management and administration, and effective communication.

The Steering Committee (SC) is the top-level decision-making body of the project, comprising one representative of each Partner Organisation, including Work Package (WP) leaders. SC members are responsible for mobilising and co-ordinating the active and coherent participation of their organisation’s team and meet 6-monthly.

The Implementation Working Group (I-WG) is responsible for ensuring successful implementation of the study. The I-WG includes representation from each hospital site, work package leads and those responsible for fidelity monitoring and project management. The I-WG are responsible for ensuring any protocol amendments are agreed and, where relevant, granted ethical approval. Given the low-risk nature of the intervention, there will be no independent safety monitoring committee. Any reported adverse events will be discussed at the monthly I-WG meetings and subsequent actions reviewed and documented. Quality control will be broken down into facets, intervention fidelity delivery, and trial recruitment and management. These will form ongoing agenda items at these meetings. HVL is the partner responsible for oversight of the study implementation. Intervention fidelity will also be monitored on an ongoing basis by Liva Healthcare through their quality assessment processes.

The External Advisory Board (EAB) will provide the B2B&Me+ consortium with external guidance on its strategic objectives and deliverables. The EAB monitor the ethical aspects of the project and account for sensitive data management and privacy concerns. The EAB will perform the role of Trial Steering Committee, and any safety concerns will be communicated to them for independent evaluation and recommended action.

### Ethical approval

Primary research ethics approval has been obtained from the National Maternity Hospital and University College Dublin in Dublin, Ireland (15^th^ April 2025: EC12.2025). Site specific study approval has also been obtained from each study site’s relevant human research ethics boards as follows:

- Granada: Bioethical Committee for Clinical Research of San Cecilio University Clinical and Mother-Infant Hospitals of Granada (Spain) (5^th^ October 2025: 281103).
- Poland: Bioethics Committee at the Pomeranian Medical University in Szczecin (25^th^ June 2025: KB-006/68/2025).
- Norway: Regional Committee for Medical and Health Research Ethics South-East Norway (16th October 2025 & 13^th^ February 2026: 941859).

Subsequent to these approvals, minor changes were made to the date of commencement and completion of recruitment at each of the 4 sites, all of which were approved by the ethics boards.

The Inclusivity in Global Research questionnaire (Supplementary Files) provides further information on ethical, cultural, and scientific considerations for this study specific to inclusivity in global research.

## Discussion

This protocol outlines a multi-country, hybrid type 3 implementation effectiveness study evaluating the integration of the B2B&Me+ programme into routine maternity care. This study will address a key gap in understanding how to translate evidence from clinical trials into routine practice and sustain interventions in real-world healthcare systems. Implementation outcomes will be prioritised while concurrently assessing effectiveness within diverse clinical contexts. The hybrid type 3 design brings a primary emphasis on implementation outcomes, while examining effectiveness in relation to intervention uptake and fidelity. This pragmatic approach enhances both internal and external validity and policy relevance.

The study is further strengthened by its theoretical grounding in the RE-AIM framework, which will guide the evaluation of reach, effectiveness, adoption, implementation and maintenance. In particular, the inclusion of adoption and maintenance outcomes will provide important insights into the sustainability and scalability of the intervention beyond the study period. The integration of qualitative methods and quantitative measures will allow for exploration of contextual determinants, supporting a more nuanced understanding of how and why implementation succeeds or falls short across settings.

The multi-country design strengthens the study by enabling the examination of implementation across heterogeneous maternity healthcare systems, organisational structures, and cultural contexts. Such cross-contextual evaluation is critical for obtaining and advancing generalisable knowledge in implementation science. By incorporating contextual mapping and stakeholder engagement, the study will identify key barriers and facilitators at multiple levels, including participant, provider and system levels. These findings will inform and benefit the design of a European implementation toolkit, which has the potential to support the transferability and adaptation of the intervention across different settings.

The evaluation of different referral strategies provides an additional level of innovation to optimise intervention reach and engagement. By systematically comparing point-of-care and more passive referral approaches, with and without follow-up contact, the study will generate evidence on mechanisms influencing participation. Understanding how referral processes affect uptake is critical, particularly for preventive interventions, where engagement may often be considered suboptimal.

Despite these strengths, several limitations should be acknowledged. The non-randomised ABA design introduces the potential for temporal confounding and limits causal inference compared to fully randomised designs. Variability in usual care across sites may also influence observed differences in outcomes. Although analytical strategies will seek to account for these factors, residual confounding cannot be excluded. Additionally, relying on a digital health platform may introduce selection bias, as participation requires engagement with smartphone technology, potentially excluding more vulnerable populations. Limiting inclusion to women speaking the country’s language also excludes more vulnerable populations. We would hope to include these populations should the use of the app be further expanded.

Implementation challenges are also anticipated, including variability in organisational readiness, competing clinical demands and differences in infrastructure across sites. Achieving an appropriate balance between intervention fidelity and context-specific adaptation will be essential, particularly in a multi-country context. While training, monitoring and process evaluation are incorporated to mitigate these challenges, variation in implementation is likely and should be viewed as both a limitation and an important, active source of learning. Furthermore, sustaining participant engagement with mHealth coaching across the perinatal period may prove challenging and entail implications for both implementation and effectiveness outcomes.

The findings from this study will have significant relevance and implications for both practice and policy. In providing evidence on the feasibility, acceptability, and cost-effectiveness of integrating a digitally supported health behaviour intervention into routine maternity care, the study will inform decision-making regarding scalability and sustainability. The development of an implementation toolkit, based on data captured across multiple European contexts, will provide practical guidance for healthcare systems seeking to incorporate interventions for GDM and related conditions.

In conclusion, this study will contribute to the advancement of implementation science by providing rigorous, informed evidence on the processes and outcomes associated with integrating an evidence-based intervention into routine maternity care. Through its hybrid design, multi-level evaluation, and cross-context scope, the B2B&Me+ project is well-positioned to generate transferable insights that can inform the implementation of interventions in maternal health and beyond. Recruitment has already commenced, with Block A1 beginning in December 2025, and overall (Blocks A2 & B) recruitment scheduled to conclude in August 2026.

### Patient and public involvement

Women with a recent history of obesity/overweight or GDM and pregnancy will be invited to join the advisory panel of experts by lived experience. This panel will form the Bump2Baby and Me+ patient and public involvement (PPI) group. The PPI panel will comprise 3–4 representatives from each site who will attend an online meeting twice a year conducted in English that will last a maximum of 1 hour. Members are expected to remain on the panel for the duration of the project. The panel can advise on all participant-facing aspects of the project, with the aim of ensuring the voice of the woman remains at the centre of the project. UCD will maintain and provide administrative support to the PPI panel.

## Contributions to the literature

- B2B&Me + is the first multi-site European study testing how a mobile health coaching programme for women at-risk of gestational diabetes can be delivered within routine maternity care across four countries.
- The study tests four strategies for inviting women to join the programme, with the intent to identify which strategy most effectively reaches and engages them in practice.
- Using established implementation science frameworks, B2B&Me+ examines what drives or hinders delivery across four diverse European health systems, generating evidence applicable beyond the study settings.
- Findings will directly inform a practical European toolkit to support maternity services in adopting the programme at scale.

## Abbrevation:

GDM: gestational diabetes mellitus
MMLGDST: monash machine learning GDM screening tool
T2D: type 2 diabetes
BMI: body mass index
GWG: gestational weight gain
B2B&Me: bump to baby and me
POC: point of care
mHealth: mobile health
EPIS framework: exploration, preparation, implementation, sustainment framework
NPT: normalisation process theory
RE-AIM framework: reach, effectiveness, adoption, implementation, maintenance framework
CEA: cost-effectiveness analysis
ICER: incremental cost-effectiveness ratio
BIA: budget impact analysis
ANOVA: analysis of variance
GEE: generalised estimating equations
PPI: patient and public involvement
WHO: World Health Organisation
FIGO: international federation of gynaecology and obstetrics
PCOS: polycystic ovary syndrome
AE: adverse event
SOP: standard operating procedure
CRF: case report form

## References

[1] Retnakaran M, Viana LV, Kramer CK. Lifestyle intervention for the prevention of type 2 diabetes in women with prior gestational diabetes: A systematic review and meta-analysis. Diabetes Obes Metab. 2023;25(5):1196–202. doi: 10.1111/dom.14966 36594235

[2] International Diabetes Federation. Diabetes Atlas. 2025.

[3] Vounzoulaki E, Khunti K, Abner SC, Tan BK, Davies MJ, Gillies CL. Progression to type 2 diabetes in women with a known history of gestational diabetes: systematic review and meta-analysis. BMJ. 2020;369:m1361. doi: 10.1136/bmj.m1361 32404325 PMC7218708

[4] Zhang C, Ning Y. Effect of dietary and lifestyle factors on the risk of gestational diabetes: review of epidemiologic evidence. Am J Clin Nutr. 2011;94(6 Suppl):1975S-1979S. doi: 10.3945/ajcn.110.001032 21613563 PMC3364079

[5] Yen IW, Lee CN, Lin MW, Fan KC, Wei JN, Chen KY, et al. Overweight and obesity are associated with clustering of metabolic risk factors in early pregnancy and the risk of GDM. PLoS One. 2019;14(12):e0225978. 10.1371/journal.pone.0225978 PMC6890240 31794594

[6] Robinson HE, O’Connell CM, Joseph KS, McLeod NL. Maternal outcomes in pregnancies complicated by obesity. Obstet Gynecol. 2005;106(6):1357–64. doi: 10.1097/01.AOG.0000188387.88032.41 16319263

[7] Rasmussen KM, Catalano PM, Yaktine AL. New guidelines for weight gain during pregnancy: what obstetrician/gynecologists should know. Curr Opin Obstet Gynecol. 2009;21(6):521–6. doi: 10.1097/GCO.0b013e328332d24e 19809317 PMC2847829

[8] Gaudet L, Wen SW, Walker M. The combined effect of maternal obesity and fetal macrosomia on pregnancy outcomes. J Obstet Gynaecol Can. 2014;36(9):776–84. doi: 10.1016/S1701-2163(15)30479-5 25222356

[9] Cedergren MI. Maternal morbid obesity and the risk of adverse pregnancy outcome. Obstet Gynecol. 2004;103(2):219–24. doi: 10.1097/01.AOG.0000107291.46159.00 14754687

[10] Al Mamun A, Mannan M, O’Callaghan MJ, Williams GM, Najman JM, Callaway LK. Association between gestational weight gain and postpartum diabetes: evidence from a community based large cohort study. PLoS One. 2013;8(12):e75679. doi: 10.1371/journal.pone.0075679 24348988 PMC3862846

[11] Teede HJ, Bailey C, Moran LJ, Bahri Khomami M, Enticott J, Ranasinha S, et al. Association of Antenatal Diet and Physical Activity-Based Interventions With Gestational Weight Gain and Pregnancy Outcomes: A Systematic Review and Meta-analysis. JAMA Intern Med. 2022;182(2):106–14. doi: 10.1001/jamainternmed.2021.6373 34928300 PMC8689430

[12] US Preventive Services Task Force, Davidson KW, Barry MJ, Mangione CM, Cabana M, Caughey AB, et al. Behavioral Counseling Interventions for Healthy Weight and Weight Gain in Pregnancy: US Preventive Services Task Force Recommendation Statement. JAMA. 2021;325(20):2087–93. doi: 10.1001/jama.2021.6949 34032823

[13] Cantor AG, Jungbauer RM, McDonagh M, Blazina I, Marshall NE, Weeks C, et al. Counseling and Behavioral Interventions for Healthy Weight and Weight Gain in Pregnancy: Evidence Report and Systematic Review for the US Preventive Services Task Force. JAMA. 2021;325(20):2094–109. doi: 10.1001/jama.2021.4230 34032824

[14] Takele WW, Vesco KK, Josefson J, Redman LM, Hannah W, Bonham MP, et al. Effective interventions in preventing gestational diabetes mellitus: A systematic review and meta-analysis. Commun Med (Lond). 2024;4(1):75. doi: 10.1038/s43856-024-00491-1 38643248 PMC11032369

[15] O’Reilly S, McAuliffe F, Teede H, Burden C, Campoy C, Maindal H, et al. Personalised mobile health coaching from early pregnancy to 12 months postpartum for women at risk of gestational diabetes: The Bump2Baby and Me multicentre randomised controlled trial. Lancet Digital Health. 2026.

[16] Craig A, Lawford H, Miller M, Chen-Cao L, Woods L, Liaw S-T, et al. Use of Technology to Support Health Care Providers Delivering Care in Low- and Lower-Middle-Income Countries: Systematic Umbrella Review. J Med Internet Res. 2025;27:e66288. doi: 10.2196/66288 40533075 PMC12223456

[17] Glasgow RE, Vogt TM, Boles SM. Evaluating the public health impact of health promotion interventions: the RE-AIM framework. Am J Public Health. 1999;89(9):1322–7. doi: 10.2105/ajph.89.9.1322 10474547 PMC1508772

[18] Gaglio B, Shoup JA, Glasgow RE. The RE-AIM framework: a systematic review of use over time. Am J Public Health. 2013;103(6):e38-46. doi: 10.2105/AJPH.2013.301299 23597377 PMC3698732

[19] Moullin JC, Dickson KS, Stadnick NA, Rabin B, Aarons GA. Systematic review of the Exploration, Preparation, Implementation, Sustainment (EPIS) framework. Implement Sci. 2019;14(1):1. doi: 10.1186/s13012-018-0842-6 30611302 PMC6321673

[20] Aarons GA, Hurlburt M, Horwitz SM. Advancing a conceptual model of evidence-based practice implementation in public service sectors. Adm Policy Ment Health Ment Health Serv Res. 2011;38(1):4–23 10.1007/s10488-010-0327-7 PMC3025110 21197565

[21] Monash Centre for Health Research and Implementation. Personal GDM for lifestyle intervention: A Web-Based, Data-Driven Prediction Tool. 2025. Available from: https://lifestyle.personalgdm.com/

[22] Porter G, Michaud TL, Schwab RJ, Hill JL, Estabrooks PA. Reach Outcomes and Costs of Different Physician Referral Strategies for a Weight Management Program Among Rural Primary Care Patients: Type 3 Hybrid Effectiveness-Implementation Trial. JMIR Form Res. 2021;5(10):e28622. doi: 10.2196/28622 34668873 PMC8567148

[23] May C, Finch T. Implementing, embedding, and integrating practices: an outline of normalization process theory. Sociology. 2009;43(3):535–54.

[24] Chan A-W, Boutron I, Hopewell S, Moher D, Schulz KF, Collins GS, et al. SPIRIT 2025 statement: updated guideline for protocols of randomised trials. Lancet. 2025;405(10491):e19–27. doi: 10.1016/S0140-6736(25)00770-6 42290521

[25] Pinnock H, Barwick M, Carpenter CR, Eldridge S, Grandes G, Griffiths CJ, et al. Standards for Reporting Implementation Studies (StaRI) Statement. BMJ. 2017;356:i6795. doi: 10.1136/bmj.i6795 28264797 PMC5421438

[26] Landes SJ, McBain SA, Curran GM. Reprint of: An introduction to effectiveness-implementation hybrid designs. Psychiatry Res. 2020;283:112630. doi: 10.1016/j.psychres.2019.112630 31722790

[27] Medisana GmbH. BS 444 connect body analysis scale.

[28] Bandura A. Social cognitive theory of self-regulation. Organ Behav Hum Decis Process. 1991;50:248–81.

[29] Ryan RM, Deci EL. Self-determination theory and the facilitation of intrinsic motivation, social development, and well-being. Am Psychol. 2000;55(1):68–78. doi: 10.1037//0003-066x.55.1.68 11392867

[30] Prochaska JO, DiClemente CC. Transtheoretical therapy: Toward a more integrative model of change. Psychotherapy Theor Res Pract. 1982;19(3):276–88. doi: 10.1037/h0088437

[31] Re-AIM. Why Use Re-AIM? Available from: https://re-aim.org/learn/why-use-re-aim/

[32] Cronin J, Gritz RM, Eisman A, Panattoni L, Ritzwoller D, Wagner N, et al. A costing guidebook for implementation scientists. Colorado Implementation Science in Cancer Control Center (COISC3); 2023.

[33] Gold MR. Cost-effectiveness in health and medicine. Oxford University Press; 1996.

[34] Raghavan R, Brownson R, Colditz G, Proctor E. The role of economic evaluation in dissemination and implementation research. Dissemination and implementation research in health: Translating science to practice. 2012. p. 113.

[35] Gold HT, McDermott C, Hoomans T, Wagner TH. Cost data in implementation science: categories and approaches to costing. Implement Sci. 2022;17(1):11. doi: 10.1186/s13012-021-01172-6 35090508 PMC8796347

[36] Eisman AB, Kilbourne AM, Dopp AR, Saldana L, Eisenberg D. Economic evaluation in implementation science: Making the business case for implementation strategies. Psychiatry Res. 2020;283:112433. doi: 10.1016/j.psychres.2019.06.008 31202612 PMC6898762

[37] Eisman AB, Quanbeck A, Bounthavong M, Panattoni L, Glasgow RE. Implementation science issues in understanding, collecting, and using cost estimates: a multi-stakeholder perspective. Implement Sci. 2021;16(1):75. doi: 10.1186/s13012-021-01143-x 34344411 PMC8330022

[38] Burt T, Button K, Thom H, Noveck R, Munafò MR. The burden of the “false‐negatives” in clinical development: analyses of current and alternative scenarios and corrective measures. Clin Transl Res. 2017;10(6):470–9. 10.1111/cts.12478 PMC6402187 28675646

[39] European Medicines Agency. Guideline on missing data in confirmatory clinical trials. 2010.

